# Structure of Aichi Virus 1 and Its Empty Particle: Clues to Kobuvirus Genome Release Mechanism

**DOI:** 10.1128/JVI.01601-16

**Published:** 2016-11-14

**Authors:** Charles Sabin, Tibor Füzik, Karel Škubník, Lenka Pálková, A. Michael Lindberg, Pavel Plevka

**Affiliations:** a Structural Virology, Central European Institute of Technology, Masaryk University, Brno, Czech Republic; b Department of Chemistry and Biomedical Sciences, Linnaeus University, Kalmar, Sweden; Instituto de Biotecnologia/UNAM

## Abstract

*Aichi virus 1* (AiV-1) is a human pathogen from the Kobuvirus genus of the Picornaviridae family. Worldwide, 80 to 95% of adults have antibodies against the virus. AiV-1 infections are associated with nausea, gastroenteritis, and fever. Unlike most picornaviruses, kobuvirus capsids are composed of only three types of subunits: VP0, VP1, and VP3. We present here the structure of the AiV-1 virion determined to a resolution of 2.1 Å using X-ray crystallography. The surface loop puff of VP0 and knob of VP3 in AiV-1 are shorter than those in other picornaviruses. Instead, the 42-residue BC loop of VP0 forms the most prominent surface feature of the AiV-1 virion. We determined the structure of AiV-1 empty particle to a resolution of 4.2 Å using cryo-electron microscopy. The empty capsids are expanded relative to the native virus. The N-terminal arms of capsid proteins VP0, which mediate contacts between the pentamers of capsid protein protomers in the native AiV-1 virion, are disordered in the empty capsid. Nevertheless, the empty particles are stable, at least *in vitro*, and do not contain pores that might serve as channels for genome release. Therefore, extensive and probably reversible local reorganization of AiV-1 capsid is required for its genome release.

**IMPORTANCE** Aichi virus 1 (AiV-1) is a human pathogen that can cause diarrhea, abdominal pain, nausea, vomiting, and fever. AiV-1 is identified in environmental screening studies with higher frequency and greater abundance than other human enteric viruses. Accordingly, 80 to 95% of adults worldwide have suffered from AiV-1 infections. We determined the structure of the AiV-1 virion. Based on the structure, we show that antiviral compounds that were developed against related enteroviruses are unlikely to be effective against AiV-1. The surface of the AiV-1 virion has a unique topology distinct from other related viruses from the Picornaviridae family. We also determined that AiV-1 capsids form compact shells even after genome release. Therefore, AiV-1 genome release requires large localized and probably reversible reorganization of the capsid.

## INTRODUCTION

*Aichi virus 1* (AiV-1) is a human pathogen from the genus Kobuvirus of the family Picornaviridae. The first strain of AiV-1 A846/88 was isolated from a patient with acute gastroenteritis in Japan in 1989 (1). Subsequently, AiV-1 was also identified in other Asian countries (2), Europe (3, 4), America (5), and Africa (6). In environmental survey studies, AiV-1 is detected with higher frequency and greater abundance than other human enteric viruses (7). Accordingly, 80 to 95% of adults worldwide have antibodies against AiV-1 (5, 7, 8). Symptoms associated with AiV-1 include diarrhea, abdominal pain, nausea, gastroenteritis, vomiting, and fever (1, 7). However, the infections can also be asymptomatic or cause only subclinical symptoms.

The AiV-1 genome is an ∼8,400-nucleotide, single-stranded, positive-sense RNA that contains one open reading frame encoding a 2,433-residue polyprotein (9). The polyprotein is cotranslationally and posttranslationally cleaved into leader protein (L-protein), viral capsid proteins VP0, VP3, and VP1, and nonstructural proteins that control the replication of AiV-1 in the infected cell (9). The cleavage is performed by virus-encoded proteases. L-protein is an additional N-terminal peptide present in some picornaviruses that can have a protease activity or fulfill another function in the virus life cycle. The capsid proteins originating from one polyprotein precursor constitute a protomer, which is the basic building block of the picornavirus capsid. While details of the assembly of kobuviruses are unknown, the assembly of related enteroviruses has been studied extensively. Initially, the VP0-VP3-VP1 protomers associate into pentamers (10, 11). Subsequently, 12 pentamers self-assemble into empty capsids (12, 13). The mechanism of self-assembly and the function of the empty capsids in the formation of enterovirus virions *in vivo*, however, are not well understood. It was shown that active replication and translation of the poliovirus genome is required for virion formation (14). RNA-containing enterovirus particles mature to virions by the cleavage of VP0 into VP2 and VP4 (15). VP4 subunits are peptides 70 to 80 residues long attached to the inner face of the capsid. Although the virions of mature enteroviruses and many other picornaviruses contain VP2 and VP4, VP0 cleavage does not occur in kobuviruses, which retain intact VP0 in mature particles. Negative-stain electron microscopy showed that AiV-1 virions have an average diameter of 30 nm (1). Furthermore, it was suggested that the AiV-1 capsid proteins form surface protrusions, giving the virus a shape similar to those of astroviruses (1, 16).

Details of the mechanism of kobuvirus genome release are not known. However, enteroviruses are also studied as models for genome release and delivery (17–21). The surface of the enterovirus capsid contains circular depressions around icosahedral 5-fold symmetry axes called “canyons.” The canyons of many enteroviruses, including major group HRVs, are the binding sites of receptors from the immunoglobulin superfamily (22–25). Nevertheless, other enteroviruses use different regions of their capsids to recognize their receptors (26). Before genome release, enterovirus virions convert into “altered” (A) particles characterized by radial expansion of the capsid and the formation of pores at the icosahedral 2-fold symmetry axes (18–21, 27–31). This conversion into A particles may be induced by binding to receptors or by exposure to the low pH of late endosomes (17, 28, 32–34). In poliovirus and coxsackievirus-A16 (CVA16), the genome release is accompanied by the exposure of the N-terminal region of VP1 and the release of the myristoylated VP4 (28, 35, 36). The VP4 subunits and N termini of VP1 were proposed to interact with the endosomal membranes and allow delivery of the RNA genome into the cytoplasm (28). Kobuviruses lack the separate capsid protein VP4. However, similar to the VP4 of enteroviruses, the N terminus of AiV-1 VP0 contains a myristoylation signal and might therefore supplement the membrane-disrupting function of VP4 (9). Here, we present a crystal structure of the AiV-1 virion and a cryo-electron microscopy (cryo-EM) reconstruction of an empty AiV-1 particle that show functionally important differences from the previously studied picornaviruses.

## MATERIALS AND METHODS

### Virus production and purification.

African green monkey kidney cells (ATCC CCL-81) grown in 50 150-mm-diameter plates in minimal essential medium (MEM) with 10% fetal bovine serum (FBS) at 37°C in a 5% CO_2_ incubator to 80% confluence were used for AiV-1 infection. The medium was aspirated from the plates, and the cells were washed with 5 ml of serum-free MEM. The cells were infected with 2 ml of the virus diluted to obtain a multiplicity of infection (MOI) of 0.2 in serum-free MEM and incubated for 3 h at 37°C in a 5% CO_2_ incubator with gentle shaking every 30 min. Subsequently, 18 ml of MEM, supplemented with 10% FBS and 1 mM l-glutamine, was added to each plate, followed by incubation at 37°C. After 72 h, a cytopathic effect was observed, and the cells were pelleted by centrifugation at 9,000 rpm at 4°C for 15 min. The virus was precipitated overnight at 4°C by adding PEG 8000 and NaCl to final concentrations of 10% and 0.5 M, respectively. The solution was centrifuged at 9,000 rpm at 4°C for 10 min, and the supernatant was discarded. The resulting pellet was resuspended in 10 ml of 20 mM HEPES (pH 7.5) and 150 mM NaCl at 4°C and then homogenized with a Dounce tissue grinder. DNase and RNase were added to final concentrations of 10 μg/ml, and the solution was incubated at 37°C for 30 min. Subsequently, trypsin was added to a final concentration of 80 μg/ml, and the solution was incubated at 37°C for an additional 30 min, followed by centrifugation at 4,500 rpm at 10°C for 10 min. The clarified supernatant was layered over a 25% (wt/vol) sucrose cushion and centrifuged in a Beckman Ti 50.2 rotor at 48,000 rpm at 10°C for 2 h. After centrifugation, the supernatant was discarded, the pellet was resuspended in 2 ml of 20 mM HEPES (pH 7.5) and 150 mM NaCl buffer at 4°C, and the virus suspension was layered onto a 10 to 40% potassium tartrate gradient and centrifuged in an SW40 rotor at 36,000 rpm at 10°C for 90 min. The gradient layer containing the virus was collected by piercing the wall of the tube with a syringe and needle. The virus-containing fraction was transferred to 20 mM HEPES (pH 7.5) and 150 mM NaCl buffer using sequential centrifugations, and buffer additions in Vivaspin (Sigma-Aldrich) columns at 4°C.

### AiV-1 crystallization, diffraction data collection, and structure determination.

AiV-1 crystallization, diffraction data collection, and structure determination were described previously (37).

### Preparation of empty AiV-1 particles by heating.

The stability of AiV-1 was determined as the temperature at which 50% of its RNA genome was accessible to fluorescent RNA-binding dye Sybr green II. Virions at a concentration of 0.02 mg/ml were incubated with Sybr green II (3,000× diluted from the stock solution according to the manufacturer's instructions), and the mixture was heated from 25 to 95°C in 1°C increments with a 2-min incubation time at each temperature in a real-time PCR instrument (Roche LightCycler 480). The fluorescence signal increases as the dye interacts with RNA that is released from the thermally destabilized particles, or the dye might be able to enter the particles. The thermal stability of the virus was estimated as the temperature corresponding to an increase in the fluorescence to 50% of the maximal value obtained when all virions were thermally denatured. The measurements were carried out in triplicates.

### Single particle data acquisition and image processing.

A solution of freshly purified AiV-1 (50 μl at concentration 2 mg/ml) was heated to 53°C for 10 min, and 3.5 μl of this sample was then immediately applied onto holey carbon grids (Quantifoil R2/1, mesh 300; Quantifoil Micro Tools) and vitrified by being plunged into liquid ethane using an FEI Vitrobot Mark IV. Grids with the vitrified sample were transferred to an FEI Titan Krios electron microscope operated at 300 kV aligned for parallel illumination in nanoprobe mode. The column of the microscope was kept at −196°C. Images were recorded with an FEI Falcon II direct electron detection camera under low-dose conditions (20 e^−^/Å^2^) with underfocus values ranging from 1.0 to 3.0 μm at a nominal magnification of 47,000, resulting in a pixel size of 1.73 Å/pixel. Each image was recorded in movie mode with 1-s total acquisition time and saved as seven separate movie frames. In total, 3,893 micrographs were acquired. The frames from each exposure were aligned to compensate for drift and beam-induced motion during image acquisition using the program SPIDER (38).

### Icosahedral reconstruction of the AiV-1 empty particles.

Regions comprised of the AiV-1 empty particles (324×324 pixels) were extracted from the micrographs using the program e2boxer.py from the package EMAN2 (39), resulting in 11,606 particles. Contrast transfer function (CTF) parameters of incoherently averaged particles from each micrograph were automatically estimated using the program ctffind4 (40). Subsequently, the particles were separated into two half-data sets for all of the subsequent reconstruction steps to follow the gold standard procedure for resolution determination (41). The structure of AiV-1 determined by X-ray crystallography was used to initiate the reconstruction. The phases of the initial model were randomized beyond a resolution of 40 Å. The images were processed using the package RELION (42). The data set of 11,606 particles was subjected to multiple rounds of two-dimensional (2D) classification and 3D classification, resulting in a near-homogeneous set of empty AiV-1 particles. Subsequent refinement was performed using the 3dautorefine procedure, with the starting model from the previous reconstruction low-pass filtered to a resolution of 60 Å. The reconstruction was followed by another round of 3D classification, where the alignment step was omitted and the estimated orientations and particle center positions from the previous refinement step were used. The resulting map was masked with a threshold mask and B-factor sharpened (43). The resulting resolution was determined at the 0.143 Fourier shell correlation of the two independent reconstructions.

### Cryo-EM structure determination and refinement.

The initial model, derived from the native AiV-1 structure obtained by X-ray crystallography (44), was fitted into the B-factor-sharpened cryo-EM map and subjected to manual rebuilding using the programs Coot and O, and coordinate and B-factor refinement using the programs CNS and phenix (45–48).

### Data analysis.

The volumes of the particles were calculated using the programs Mama and Voidoo of the Uppsala Software Factory (49). Average radii were calculated using the program Moleman2 from the Uppsala Software Factory (50). Figures were generated using the programs UCSF Chimera (51) and PyMOL (The PyMOL Molecular Graphics System, v1.7.4; Schrödinger, LLC). Structure-based alignments of biological protomers of various picornaviruses were prepared using the Multiseq tool in VMD and the program STAMP (52). The root mean square deviation (RMSD) values provided by STAMP were used to create a nexus-format matrix file, which was converted into a structure-based phylogenetic tree and visualized using the program FigTree (http://tree.bio.ed.ac.uk/software/figtree/).

### Accession number(s).

The Protein Data Bank (PDB) model of native AiV-1, together with structure factor amplitudes and phases derived by phase extension, was deposited under PDB code 5AOO. A cryo-EM reconstruction map of the empty AiV-1 particle was deposited with EMDB under the number EMD-4112, and the fitted coordinates were deposited under PDB code 5LVC.

## RESULTS AND DISCUSSION

### Structure of AiV-1 virion and capsid proteins.

**FIG 1:**
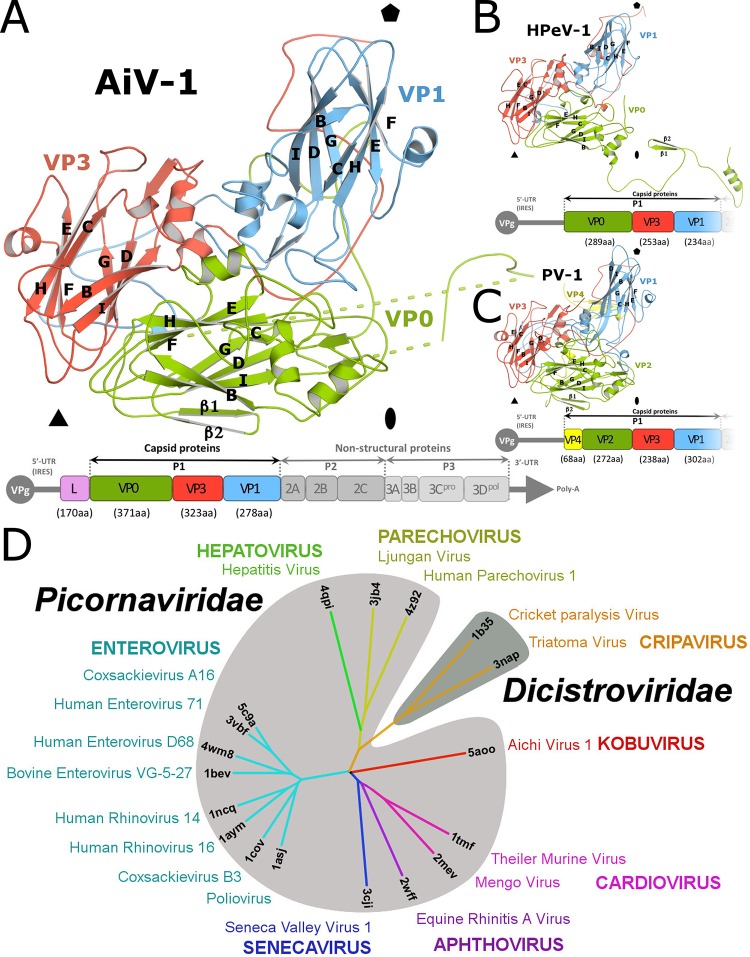
Structure of icosahedral asymmetric unit of AiV-1 and structure-based evolutionary tree of picornaviruses and dicistroviruses. (A to C) Cartoon representations of icosahedral asymmetric units of AiV-1 (A), HPeV-1 (B), and poliovirus 1 (C). VP1 subunits are shown in blue, VP2 and VP0 are shown in green, VP3 is shown in red, and VP4 is shown in yellow. Schematic diagrams of the genome organizations of the viruses are shown below the structure figures. (D) Phylogenetic tree based on the structural similarity of icosahedral asymmetric units of indicated viruses from the Picornaviridae and Dicistroviridae families. For details on the construction of the diagram, please see Materials and Methods.

The crystal structure of the AiV-1 virion was determined to a resolution of 2.1 Å using X-ray crystallography. The diffraction data were affected by perfect hemihedral twinning. The structure determination process has been described elsewhere (37), but for completeness of the discussion the basic crystallographic and structure quality indicators are reprinted here (Table 1). The maximum outer diameter of the AiV-1 capsid is 318 Å. The capsid is built from major capsid proteins VP0, VP1, and VP3 arranged with pseudo-T3 icosahedral symmetry (Fig. 1A). VP1 subunits form pentamers around 5-fold axes, while VP0 and VP3 alternate around the icosahedral 3-fold axes. The three capsid proteins have jellyroll β-sandwich folds with β-strands named according to the picornavirus convention B to I (53). Two antiparallel β-sheets forming the cores of the subunits contain strands BIDG and CHEF, respectively (Fig. 1A). Loops of the capsid proteins are named according to the β-strands they connect. N termini of the major capsid proteins are located on the inside of the capsid, whereas C termini are exposed at the virion surface. A complete model of the AiV-1 icosahedral asymmetric unit could be built apart from residues 1 to 12, 56 to 63, and 76 to 111 of VP0, residues 84 to 87 and 234 to 253 of VP1, and residues 221 to 223 of VP3.

**TABLE 1:** AiV-1 virion and empty particle structure quality indicators

Parameter	AiV-1 empty particle cryo-EM structure	AiV-1 virion crystal structure^*b*^
Space group	NA^*d*^	I23
Unit cell dimensions		
*a*, *b*, *c* (Å)	NA	350.80, 350.80, 350.80
α, β, γ (°)	NA	90, 90, 90
Resolution (Å)	4.2	72.0–2.3 (2.34–2.30)
*R*_merge_^*a*^	NA	0.15 (0.63)
〈*I*〉/〈σ*I*〉	NA	6.0 (1.8)
Completeness (%)	NA	91.0 (94.0)
Observation multiplicity	NA	3.2 (3.0)
No. of observations	NA	902,384 (43,198)
No. of unique observations	NA	282,853 (14,471)
*R*_work_	0.34	0.33^*e*^
No. of:		
Protein atoms*	5,455	5,791
Water atoms*	NA	147
RMSD		
Bond lengths (Å)	0.016	0.013
Bond angles (°)	1.57	1.43
Ramachandran statistics (%)^*c*^		
Preferred regions	89.1	95.7
Allowed regions	8.4	4.0
Disallowed regions	2.5	0.3
Avg atomic B factor (Å^2^)	127.8	22.9

a *R*_merge_ = Σ_h_Σ_j_|l_hj_ − 〈l_h_〉|/ΣΣ|l_hj_|. *, Statistics are given for one icosahedral asymmetric unit.

b Statistics for the highest-resolution shell are indicated in parentheses.

c Determined according to the criterion of Molprobity (78).

d NA, not applicable.

e All reflections were used in the refinement. The *R_free_* value, if it were calculated, would be very similar to R_work_ because of the 5-fold noncrystallographic symmetry present in the crystal. Therefore, the *R*_free_ would not provide an unbiased measure of model quality in this case (55).

### Topology of AiV-1 virion surface is distinct from the previously structurally characterized picornaviruses.

**FIG 2:**
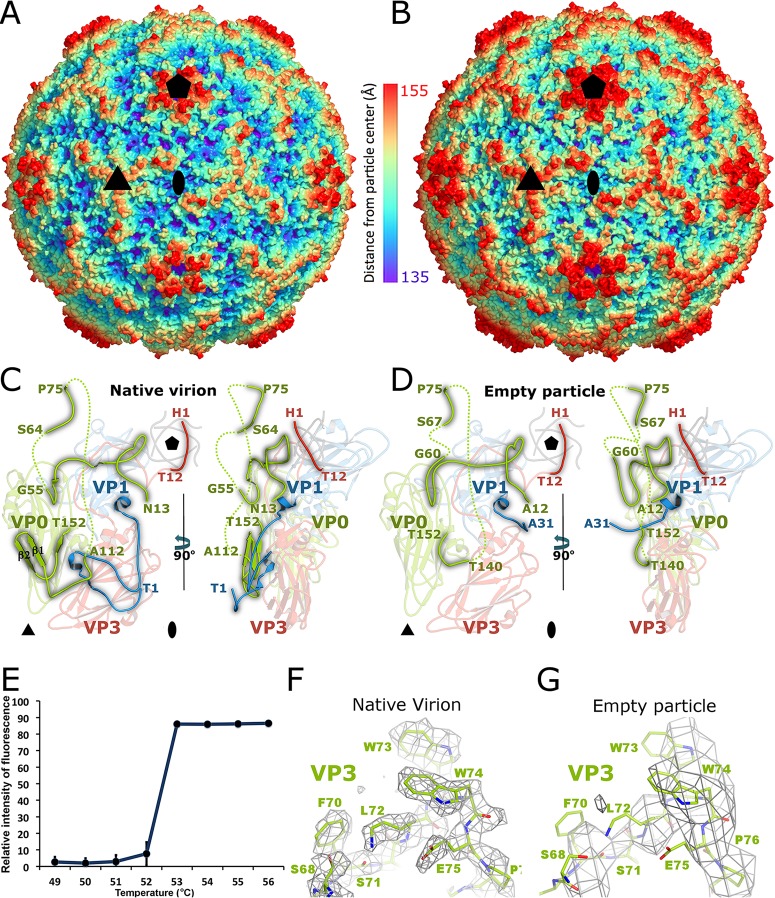
Changes of AiV-1 capsid associated with genome release. (A and B) Surface rendering of AiV-1 virion (A) and empty particle (B) rainbow-colored based on distance from the particle center. The positions of selected 5-fold, 3-fold, and 2-fold icosahedral symmetry axes are indicated with pentagons, triangles, and ovals, respectively. (C and D) Icosahedral asymmetric unit of native AiV-1 virion (C) and the empty particle (D). VP1, VP0, and VP3 are shown in blue, green, and red, respectively. N-terminal arms of the capsid proteins are highlighted in brighter colors. The positions of the 5-fold-symmetry-related N termini of VP3 subunits are shown in gray. Dashed lines indicate the putative positions of the unstructured chains. The positions of 5-fold, 3-fold, and 2-fold icosahedral symmetry axes are indicated with pentagons, triangles, and ovals, respectively. (E) A Sybr green fluorescence assay was performed to measure the stability of AiV-1 virions. AiV-1 virions were mixed with Sybr green dye II and heated to the indicated temperatures (*x* axis). The fluorescence signal increases as the dye binds to RNA that is released from thermally destabilized particles. Error bars indicate the standard deviations of the measurements. Please see Materials and Methods for details. (E and F) Examples of electron densities of AiV-1 virion at a resolution of 2.1 Å (E) and empty particle at the resolution of 4.2 Å (F). The maps are contoured at 1.5σ.

**FIG 3:**
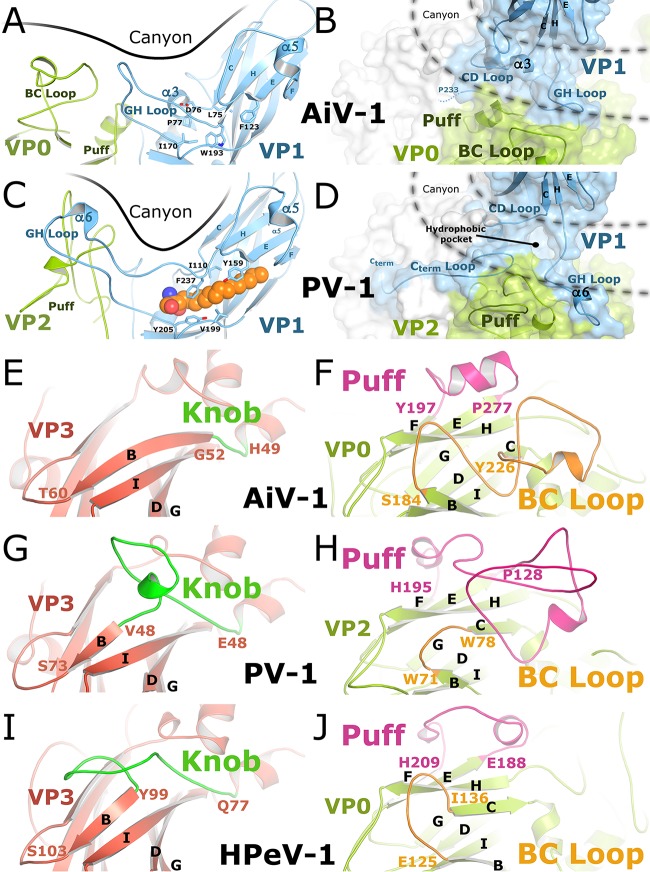
Comparison of capsid surface features of AiV-1, poliovirus 1, and HPeV-1. (A to D) Side and top views of the canyons of AiV-1 (A and B) and poliovirus 1 (C and D). Capsid proteins are shown in a cartoon representation. VP1 is shown in blue, and VP2/VP0 are shown in green. A pocket factor of poliovirus 1 is shown in space-filling representation in orange. Residues that interact with the pocket factor are depicted as sticks. The molecular surface is displayed in panels B and D. (E to J) Comparison of subunits VP0 and VP3 of AiV-1 (E and F), poliovirus 1 (G and H), and HPeV-1 (I and J). VP3 of AiV-1 (E) lacks the loop knob (highlighted in green) that forms a prominent surface feature of poliovirus 1 (G) and HPeV-1 (I). The loop puff (highlighted in magenta) of AiV-1 (F) contains a single 7-residue helix and is shorter than the puffs of poliovirus 1 (H) and HPeV-1 (J). The BC loop (highlighted in orange) of AiV-1 forms a prominent feature in its capsid and is longer than those of poliovirus 1 (H) and HPeV-1 (J). Selected secondary structure elements are labeled.

While structures of numerous viruses from the Picornaviridae and Dicistroviridae families have been determined previously, AiV-1 is the first characterized representative of the Kobuvirus genus. AiV-1 shares less than 26% sequence identity with poliovirus 1, HRV14, EV71, human parechovirus 1 (HPeV-1), and hepatitis virus A (Table 2) (54–57). Structure-based phylogenetic analysis indicates that AiV-1 is quite distinct from all previously determined structures of picornaviruses and dicistroviruses (Fig. 1D). Most of the differences are located at the capsid surface. Our high-resolution analysis did not confirm the presence of the previously proposed astrovirus-like protrusions in the AiV-1 capsid (1, 16). Instead, the AiV-1 virion is rather spherical in shape with plateaus around 3-fold axes, slight depressions at 2-fold axes, and low protrusions around icosahedral 5-fold axes that are encircled by circular depressions that we, according to the enterovirus convention, call canyons (Fig. 2A and B). However, the AiV-1 canyon is shallower and has a different shape to that of enteroviruses (Fig. 2C). The differences in the capsid topology between AiV-1 and other enteroviruses are due to different lengths of loops of the capsid proteins that form the capsid surface. The central wall of the enterovirus canyon is formed by CD loops of VP1, whereas the outer wall is formed by the EF loop of VP2 called puff and a loop before β-strand B of VP3 called knob, the CD loop of VP3, and the GH loop of VP1 (Fig. 3C, D, G, and H). However, capsid protein VP3 of AiV-1 lacks the knob loop and its VP0 (a homologue of enterovirus VP2) contains only a very small puff, including a single seven-residue α-helix (Fig. 3A, B, E, and F). Interestingly, the puff and knob loops of AiV-1 are shorter that those in HPeV-1, which lacks the canyon altogether (Fig. 3I and J). However, the BC loop of VP0 of AiV-1 is relatively elongated and forms a protrusion on the capsid surface located in the volume occupied by the puff and knob of enteroviruses (Fig. 3E to J). Therefore, the BC loop of AiV-1 VP1 substitutes for the missing knob and small puff of AiV-1 and forms the outer wall of the canyon (Fig. 3A, B, and F). Moreover, the GH loop of AiV-1 VP1 is relatively short and fills the canyon instead of reinforcing its outer wall, as is the case in poliovirus 1 (Fig. 3A and B). The long C-terminal arm of poliovirus 1 VP1, which wraps around a side of the puff, is not structured in AiV-1, resulting in a relative broadening of the canyon (Fig. 3A to D). Furthermore, the depth of the AiV-1 canyon is further diminished by the α3 helix in the CD loop of AiV-1 VP1 that partially fills the volume of the canyon (Fig. 3A and B). In summary, the differences in the surface-exposed loops make AiV-1 unique among picornaviruses and dicistroviruses characterized to date. They are reflected in the isolated position of AiV-1 in the structure-based evolutionary tree (Fig. 1D). The differences in surface topology indicate the possibly of a different type of receptor binding in AiV-1 than is found in enteroviruses.

**TABLE 2:** Sequence and structural similarity of capsid proteins of selected picornaviruses

Family and genus	Virus	Sequence and structural similarity (% or RMSD)^*a*^										
		AiV-1	TMEV	ERAV	SVV-1	PV-1	HRV14	EV71	CVA16	HAV	HPeV-1	CrPV
Picornaviridae												
Kobuvirus	AiV-1		1.9	2.0	1.8	1.8	1.8	1.8	1.8	2.1	2.2	2.6
Cardiovirus	TMEV	28		1.9	1.4	1.5	1.5	1.7	1.6	2.0	2.1	2.8
Aphthovirus	ERAV	25	33		1.9	1.8	1.7	1.7	1.7	2.4	2.6	3.0
Senecavirus	SVV-1	28	38	31		2.3	1.7	1.8	1.7	2.0	2.2	3.2
Enterovirus	PV-1	24	29	24	29		1.0	1.1	1.0	2.1	2.1	2.6
	HRV14	25	26	25	28	49		1.1	1.2	2.1	2.1	2.8
	EV71	26	30	23	29	44	44		0.5	2.1	2.2	2.5
	CVA16	25	30	22	30	47	44	79		2.1	2.2	2.6
Hepatovirus	HAV	17	21	18	20	18	19	17	15		2.1	2.6
Parechovirus	HPeV-1	20	20	18	18	17	17	17	15	18		2.7
Dicistroviridae												
Cripavirus	CrPV	16	14	17	11	13	11	14	14	16	17	

a The top right portion of the table presents the root mean square deviations (Å) of superimposed Cα atoms of the respective 3D structures. The distance cutoff for inclusion of residues in the calculation was 3.8 Å. Capsid protein protomers corresponding to icosahedral asymmetric units consisting of subunits VP1 to VP4 were used in the comparisons. The program Coot was used for superposition of the molecules (47). The bottom left portion of the table presents the percent identities between respective virus coat protein sequences. Gaps were ignored in the calculations.

### Absence of hydrophobic pocket in VP1.

The genome release of some enteroviruses can be initiated by the interaction of the virus with receptors with an immunoglobulin fold that bind to the canyon (25, 53, 58). At the bottom of the canyons of some enteroviruses is a small aperture leading to a hydrophobic pocket within the β-barrel core of the VP1 subunit (54, 55). The pocket is filled with a lipid moiety called a “pocket factor,” which was proposed to function in the regulation of virus stability and uncoating (25). Importantly, compounds binding to the VP1 pocket with high affinity have been demonstrated to increase enterovirus stability, block its genome release or receptor binding, and prevent virus infection (59–64). However, in comparison to enteroviruses the β-sandwich core of AiV-1 VP1 is filled with hydrophobic side chains of residues and does not form the pocket (Fig. 3A and C). In this respect, AiV-1 is similar to other picornaviruses such as cardioviruses (65–67), parechoviruses (56, 68), and aphthoviruses (32, 69). Therefore, capsid-binding inhibitors are unlikely to be effective against AiV-1 and other kobuviruses.

### Structural changes of AiV-1 capsid associated with genome release.

**FIG 4:**
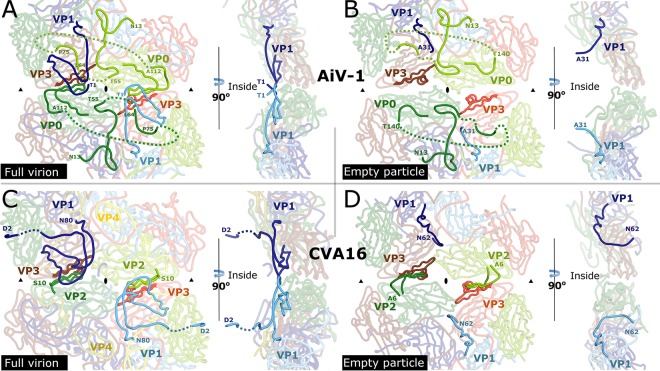
Comparison of interpentamer contacts in virions and expanded particles of CVA16 and AiV-1. (A to D) Structures of AiV-1 virion (A), AiV-1 empty particle (B), CVA16 virion (C), and CVA16 empty particle (D) in the vicinity of the icosahedral 2-fold axes viewed from the particle center (left) and perpendicular to the capsid surface (right). VP1 subunits are shown in blue, VP0/VP2 are shown in green, and VP3 is shown in red. Subunits from different icosahedral asymmetric units are distinguished by color tones. N-terminal arms of VP0 in AiV-1 and VP2 in CVA16 that participate in interpentamer contacts are shown in bright green. Dashed lines indicate the putative positions of unstructured residues. N-terminal residues of VP1 are shown in bright blue. Residues of VP3-forming strands βE and βF that interact with the N termini of VP0/VP2 are shown in bright red. The positions of icosahedral 2-fold and 3-fold symmetry axes are indicated with ovals and triangles, respectively.

**FIG 5:**
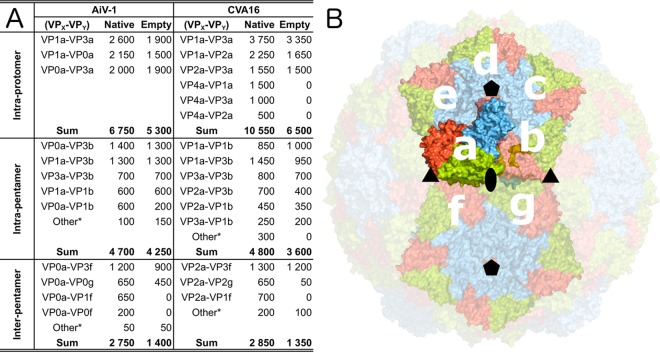
Buried surface areas of interfaces within AiV-1 and CVA16 virions and empty particles. (A) List of buried surface areas. Individual subunits are labeled according to their relative positions shown in panel B. “Other*” indicates the sum of buried surface areas of additional small interfaces. (B) Capsid surface representation of AiV-1 virion with subunits VP0, VP1, and VP3 shown in green, blue, and red, respectively. Icosahedral asymmetric units considered for buried surface calculations are labeled with letters.

It has been shown previously that virions of enteroviruses convert to A particles before the genome release (17, 28, 33, 34). The A particles are characterized by an expanded virion diameter and channels in the capsid that were speculated to serve in the release of the RNA genomes. The empty capsid shells produced after the genome release were named B particles, which are structurally similar to the A particles. It was demonstrated that the formation of A particles and the genome release of many picornaviruses might be induced nonphysiologically by heating the virions to 42 to 56°C (21). In order to study the genome release of AiV-1, its virions were gradually heated, and the genome release was monitored (Fig. 2E). AiV-1 virions released their RNA rather abruptly at 53°C. Electron microscopy of AiV-1 virions heated to 53°C for 10 min identified 95% of empty capsids, which were used to reconstruct the empty particle to a resolution of 4.2 Å (Fig. 2G and Table 1). The AiV-1 empty capsid is expanded by 7.6 Å in diameter relative to the native virus (Fig. 2A and B). The volume of the particle increases from 4.8 × 10^6^ Å^3^ to 5.5 × 10^6^ Å^3^. The structure of the empty particle differs from that of the native virion, mostly in the contacts between the pentamers of capsid protein protomers. In the native AiV-1 virion, the interpentamer contacts are mediated by strand β2 of VP0, which interacts with β-strand F of VP3 (Fig. 2C and 4A). Strands β1 and β2 of VP0 extend the β-sheet CHEF of VP3. However, in the empty particle residues 112 to 139, which form the β1 and β2 strands of VP0, are disordered (Fig. 2D and 4B). As a consequence, the interpentamer interface is reduced from 2,750 to 1,400 Å^2^ (see Fig. 5). Furthermore, residues 139 to 144 of VP0 form new interactions with the core of subunit VP3 (Fig. 4B). At the same time, residues 55 to 60 of VP0 became structured in the empty AiV-1 particle and residues 67 to 75 retain the same structure as in the native virions (Fig. 2C and D and Fig. 4A and B). Therefore, the N-terminal arm of AiV-1 VP0 does not appear to be externalized from the empty particle. This is in contrast to the presumption that the N-terminal part of AiV-1 VP0 is functionally homologous to the VP4 of other picornaviruses and might therefore play a role in the transport of the virus genome across the endosomal membrane into the host cytoplasm. However, it is possible that *in vivo*, because of interactions with an as-yet-unknown receptor, the empty AiV-1 particles dissociate into pentamers and the VP0 N termini could interact with the membranes.

The structure of HPeV-1, which also contains VP0 in mature virions, was recently determined (56). However, in contrast to AiV-1, empty particles of HPeV-1 rapidly dissociate into pentamers after the genome release (56, 70). Major differences between AiV-1 and HPeV-1 are in the interpentamer interactions mediated by the N-terminal arm of VP0 (Fig. 1A and B). While in AiV-1 the β1 and β2 strands extend the β-sheet CHEF of VP3 from the icosahedral asymmetric unit from a neighboring pentamer; in HPeV-1 the N-terminal arm forms a loop that stretches around the icosahedral 2-fold axis and the β-strands extend the β-sheet of the VP3 subunit from the same pentamer (Fig. 1A and B). The differences in the positioning of the N-terminal arm of VP0 might influence the stability of the empty capsid, since enteroviruses, which produce empty particles after genome release, have the same type of interpentamer interaction mediated by the N terminus of VP0/VP2 as that of AiV-1 (Fig. 1C).

The N-terminal arm of AiV-1 VP1 undergoes structural reorganization upon the formation of the empty particle. Residues 1 to 30 become disordered, and residues 31 to 35 refold into a new structure with the last resolved residue pointing toward the particle center (Fig. 4A and B and Fig. 2C and D). This is in contrast to the previous observation of the A particle of CVA16, in which the N terminus of VP1 was exposed at the capsid surface (Fig. 4C and D) (29). The N termini of VP1 of enteroviruses were proposed to interact with membranes and facilitate the delivery of the virus genome into the cytoplasm (29, 71). Similar to the N termini of AiV-1 VP0s, the N termini of AiV-1 VP1 subunits might interact with the membrane after particle dissociation.

The structural changes of the AiV-1 capsid linked to the genome release result in a reduction of interpentamer contacts by 49%, whereas the contacts within the protomer and within the pentamer are only reduced by 22 and 10%, respectively (Fig. 5). The reduction in the interface area cannot be directly converted to binding energies, but the reduced interpentamer contacts indicate that the expanded AiV-1 particles might be prone to the formation of capsid pores for genome release or to disassembly into pentamers.

### Differences between kobuvirus and enterovirus genome release.

The structure of the empty particle of AiV-1 is different from that of empty and A particles of enteroviruses. “A” particles of CVA7, EV71, poliovirus 1, HRV-2, and CVA16 contain two types of pores located at icosahedral 2-fold axes and between icosahedral 2-fold and 5-fold symmetry axes (Fig. 4C and D) (18–21, 27–31). In the CVA16 A particle, the pores at 2-fold axes were proposed to allow externalization of VP1 N termini that then translocate to the channels between 2-fold and 5-fold axes (Fig. 4C and D) (29). In addition, the pores at 2-fold axes were speculated to serve as channels for the release of VP4 subunits and the RNA genome. The borders of the channels located at 2-fold axes in enterovirus particles are formed by helix α3 from the CD loop of VP2 and EF loop of VP3 (29, 72). In contrast to the enterovirus A and empty particles, the empty particle of AiV-1 does not contain any pores (Fig. 2B). Aphthovirus equine rhinitis A virus (ERAV) also forms empty particles with compact capsids that do not contain any pores for genome release (32). Nevertheless, the empty particles of ERAV rapidly dissociate into pentamers.

**FIG 6:**
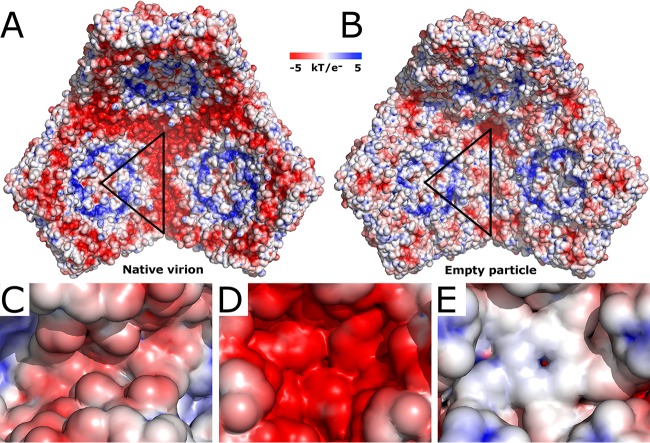
Comparison of charge distribution inside the AiV-1 virion and empty particle. (A and B) Comparison of electrostatic potential distribution inside the native virion (A) and empty particle (B). Three pentamers of capsid protein protomers are displayed. The borders of a selected icosahedral asymmetric unit are highlighted with a black triangle. (C to E) Details of the electrostatic surface of the empty particle around 2-fold (C), 3-fold (D), and 5-fold (E) symmetry axes.

The most likely positions for the formation of channels in the AiV-1 capsid are the borders of the pentamers of capsid protein protomers and specifically areas around the icosahedral symmetry axes. Helices α3 of VP0 subunits related by a 2-fold axis move 1.8 Å away from each other when the AiV-1 virion converts to the empty particle (Fig. 4A and B). Nevertheless, the movement does not result in the formation of a channel around the 2-fold axis (Fig. 2B and 4B). The channels at the 2-fold axes observed in the A and empty particles of enteroviruses are also not of sufficient size and require expansion in order to allow the genome release (Fig. 2D) (29, 71, 72). It is therefore possible that an opening in the AiV-1 capsid at the 2-fold axis might serve as a channel for genome release; however, more extensive local reorganization of the capsid than in enteroviruses would be required. This possibility is to some extent supported by the presence of residues with neutral and negatively charged side chains in the vicinity of the 2-fold symmetry axis of AiV-1, similar to the situation in enteroviruses (Fig. 6A to C) (73). The negative charge might provide a “slippery” surface facilitating the egress of viral RNA (74).

It was proposed previously that an “iris-like aperture” movement of the N termini of VP3 subunits might result in the opening of 10-Å diameter channels through the rhinovirus capsid, which might enable the exit of the genomic RNA (75, 76). The narrowest constriction along the AiV-1 5-fold axis is formed by the N-terminal arms of VP3 subunits, which might perform such an “iris-like aperture” movement (Fig. 2C). However, the structures of AiV-1 VP1 and VP3 subunits, which are in contacts around the 5-fold axes, are not affected by the capsid expansion (Fig. 2D), and the pore along the 5-fold axis is obstructed in both the native and the empty particle structures (Fig. 6A, B, and E). Therefore, the 5-fold axes of the AiV-1 capsid do not seem to be likely portals for the genome release. It is possible that the iris-like aperture movements in the rhinovirus capsids may represent structural rearrangements accompanying the genome release rather than formation of a pore through which the RNA exits the virion. Similarly, the 3-fold axes of the AiV-1 capsid are entirely closed by side chains located in their vicinity, and the internal face of AiV-1 capsid around the 3-fold axes is covered by negatively charged residues (Fig. 6B and D).

Comparison of the native virion and empty particle structures of AiV-1 indicates that the genome release necessitates the formation of a special pore within the capsid shell. However, the possibility of obtaining a 4.2-Å-resolution reconstruction of the empty particle indicates that the pore does not affect the overall icosahedral symmetry of the capsid or that the structural changes required for the genome release are reversible. In that case the empty capsids would not provide information on how the genome was released from the particle. The absence of pores in the AiV-1 capsid presents an obstacle for genome release. It was shown previously that the genome release of HRV2 proceeds in the 3′-to-5′ direction (77). Assuming that AiV-1 genome release has the same directionality, it is not clear how the 3′ end of the AiV-1 genome might induce the formation of a pore in the capsid or how it finds the special preformed pore within the capsid. The alterations in the structures of the N termini of VP2 and VP1 connected with the formation of the empty AiV-1 particle result in the removal of the negative charge that is located at the edges of the pentamers in the native virion (Fig. 6A and B). This may promote interactions of the genomic RNA with the capsid and its eventual release from the virion.
